# Supplementary material for: A Likelihood Approach for Real-Time Calibration of Stochastic Compartmental Epidemic Models
Source: PLoS Comput Biol. 2017 Jan 17;13(1):e1005257. doi: 10.1371/journal.pcbi.1005257 (PMC5240920; doi:10.1371/journal.pcbi.1005257)
Supplement: S1 File — (TAR.GZ) [file pcbi.1005257.s014.tar.gz › HSPH_Online-SI-Revision/EnKF07/n10-mild/n10-mild_PF2_EnKF-summary2.pdf]

peak -8	3w spec	3w cum	5w spec	5w cum	end	3w spec LOG	3w cum LOG	5w spec LOG	5w cum LOG	end LOG
mean	562.	1555.8	117.6	465.8	88.6	1.	0.8	1.6	0.6	0.7
median	171.2	471.2	97.	232.3	86.4	0.9	0.6	1.8	0.5	0.6
quantile .1	83.9	105.2	84.7	58.5	64.2	0.4	0.3	0.7	0.3	0.3
quantile .9	940.2	3888.6	198.3	1174.3	110.8	1.5	1.5	2.5	0.3	1.
42	24	23	30	35						

peak -4	3w spec	3w cum	5w spec	5w cum	end	3w spec LOG	3w cum LOG	5w spec LOG	5w cum LOG	end LOG
mean	111.4	181.6	96.9	123.6	89.	1.2	0.6	1.9	0.7	0.8
median	95.1	135.9	95.	106.4	91.	1.2	0.6	1.9	0.7	0.9
quantile .1	83.2	71.1	84.4	71.3	68.	0.7	0.3	1.1	0.4	0.3
quantile .9	147.	349.4	105.5	171.4	101.1	1.8	0.7	2.5	0.4	1.1
21	40	7	35	26						

peak	3w spec	3w cum	5w spec	5w cum	end	3w spec LOG	3w cum LOG	5w spec LOG	5w cum LOG	end LOG
mean	117.	157.	102.1	131.3	101.	1.3	0.6	1.7	0.7	0.7
median	104.8	125.3	98.7	109.3	98.1	1.1	0.6	1.8	0.6	0.7
quantile .1	87.	76.6	88.3	81.	80.5	0.7	0.4	0.8	0.4	0.5
quantile .9	155.5	303.	118.8	217.3	124.3	2.1	0.8	2.6	0.4	1.
22	35	10	34	28						

peak +4	3w spec	3w cum	5w spec	5w cum	end	3w spec LOG	3w cum LOG	5w spec LOG	5w cum LOG	end LOG
mean	241.6	298.4	2553.9	254.3	219.9	1.1	0.5	1.4	0.5	0.5
median	155.8	220.2	130.9	189.4	168.6	1.1	0.4	1.5	0.4	0.5
quantile .1	90.2	107.9	85.	94.8	90.6	0.4	0.3	0.8	0.3	0.3
quantile .9	251.4	521.5	384.6	354.6	306.2	1.7	0.6	1.8	0.3	0.7
42	44	34	46	46						

peak +8	3w spec	3w cum	5w spec	5w cum	end	3w spec LOG	3w cum LOG	5w spec LOG	5w cum LOG	end LOG
mean	3682.4	28 128.9	9727.7	30 191.3	34 298.6	1.	0.8	1.3	0.7	0.8
median	268.7	565.5	220.4	497.8	410.6	0.9	0.6	1.3	0.6	0.6
quantile .1	87.5	173.3	88.9	156.8	90.5	0.5	0.4	0.7	0.3	0.3
quantile .9	3586.3	3590.3	4853.	4929.9	5906.8	1.8	1.1	1.9	0.3	1.3
45	25	39	31	31	{}	{}	{}	{}	{}	{}
